# α-smooth muscle actin is not a marker of fibrogenic cell activity in skeletal muscle fibrosis

**DOI:** 10.1371/journal.pone.0191031

**Published:** 2018-01-10

**Authors:** Wanming Zhao, Xingyu Wang, Kai-Hui Sun, Lan Zhou

**Affiliations:** 1 Department of Neurology, Icahn School of Medicine at Mount Sinai, New York, New York, United States of America; 2 Department of Medicine Lung Biology Lab, University of California, San Francisco, San Francisco, California, United States of America; University of Minnesota Medical Center, UNITED STATES

## Abstract

α-Smooth muscle actin (α-SMA) is used as a marker for a subset of activated fibrogenic cells, myofibroblasts, which are regarded as important effector cells of tissue fibrogenesis. We address whether α-SMA-expressing myofibroblasts are detectable in fibrotic muscles of *mdx*^*5cv*^ mice, a mouse model for Duchenne muscular dystrophy (DMD), and whether the α-SMA expression correlates with the fibrogenic function of intramuscular fibrogenic cells. α-SMA immunostaining signal was not detected in collagen I (GFP)-expressing cells in fibrotic muscles of *ColI-GFP/mdx*^*5cv*^ mice, but it was readily detected in smooth muscle cells lining intramuscular blood vessel walls. α-SMA expression was detected by quantitative RT-PCR and Western blot in fibrogenic cells sorted from diaphragm and quadriceps muscles of the *ColI-GFP/mdx*^*5cv*^ mice. Consistent with the more severe fibrosis in the *ColI-GFP/mdx*^*5cv*^ diaphragm, the fibrogenic cells in the diaphragm exerted a stronger fibrogenic function than the fibrogenic cells in the quadriceps as gauged by their extracellular matrix gene expression. However, both gene and protein expression of α-SMA was lower in the diaphragm fibrogenic cells than in the quadriceps fibrogenic cells in the *ColI-GFP/mdx*^*5cv*^ mice. We conclude that myofibroblasts are present in fibrotic skeletal muscles, but their expression of α-SMA is not detectable by immunostaining. The level of α-SMA expression by intramuscular fibrogenic cells does not correlate positively with the level of collagen gene expression or the severity of skeletal muscle fibrosis in the *mdx*^*5cv*^ mice. α-SMA is not a functional marker of fibrogenic cells in skeletal muscle fibrosis associated with muscular dystrophy.

## Introduction

Tissue fibrosis is characterized by excessive deposition of extracellular matrix (ECM) proteins, including collagens and fibronectin. It is resulted from an uncontrolled wound-healing response to chronic tissue injury and inflammation. It can affect all tissue and organ systems, which causes considerable morbidity and mortality. The well recognized and studied fibrotic disorders include pulmonary fibrosis, liver cirrhosis, renal sclerosis, and scleroderma [1].

Duchenne muscular dystrophy (DMD) is the most common genetic muscle disease, which affects 1 in 3,500 live male births [2, 3]. It is a devastating disease characterized by progressive skeletal and cardiac muscle weakness with premature death around age 20 years [2–5]. It is caused by a defective dystrophin gene on the X chromosome. Dystrophin deficiency disrupts the dystrophin-glycoprotein complex (DGC), which normally spans muscle membrane to enable muscle to sustain mechanical stretch and contraction. Defective DGC leads to an increase of sarcolemmal permeability, influx of calcium into sarcoplasm, and activation of protease to cause myofiber necrosis and degeneration. This in turn triggers an inflammatory response for injury repair. The muscle injury associated with DMD cannot be repaired completely as the defect is genetic and chronic. The chronic muscle injury in DMD induces chronic inflammation with persistent production of pro-fibrotic cytokines by inflammatory cells, and excessive ECM protein synthesis and deposition by activated fibrogenic cells. Fibrosis is a prominent pathological feature of muscle biopsies from patients with DMD [6]. It contributes to limb, respiratory, and cardiac muscle dysfunction, and the lethal phenotype [6–8].

The most commonly used animal model for studying DMD is *mdx* mice. Muscle pathology in the *mdx* mice also features chronic inflammation and progressive fibrosis [9–13]. In the *mdx* mice, the muscle inflammation starts around 3 weeks of age. A high level of inflammation persists to 2–3 months, and then subsides spontaneously in limb muscles. Progressive muscle fibrosis predominantly occurs in diaphragm, which correlates with an impaired respiratory function [10–15]. Studies from our group and others have demonstrated that ameliorating muscle fibrosis represents a viable therapeutic approach for DMD, because it can improve muscle function and dystrophy phenotype in the DMD mouse model [16–21]. Although gene and cell therapies are very promising and may ultimately cure DMD, anti-fibrotic therapies are likely needed to improve the local tissue environment to increase the gene and cell engraftment efficiency [22–25].

In order to develop effective anti-fibrotic therapies, it is important to study fibrogenic cells, the effector cells of tissue fibrogenesis. These cells can be derived from multiple origins, including tissue resident fibroblasts, epithelial-mesenchymal transition, endothelial-mesenchymal transition, pericytes, and circulating fibrocytes [1]. Myofibroblasts are a subset of activated fibrogenic cells with increased expression of ECM proteins and neo-expression of α-smooth muscle actin (α-SMA) [26]. Myofibroblasts are conventionally identified by co-expression of collagen I and α-SMA [27–30]. α-SMA is not just a marker of myofibroblasts, it can also increase the contractile activity of myofibroblasts [31]. It has been shown that the contractile force generated by myofibroblasts contributes to the activation of integrin-bound latent transforming growth factor beta 1 (TGF-β1) [32, 33], which is a potent fibrogenic growth factor. Myofibroblasts are thus regarded as the key activated fibrogenic cells for normal wound repair and abnormal tissue fibrogenesis, and α-SMA is used as a marker for the fibrogenic activity of activated tissue fibrogenic cells [26, 34]. However, a recent study showed that α-SMA was an inconsistent marker of the fibrogenic function [35]. Myofibroblasts with the α-SMA expression did not play an important role in the lung fibrosis induced by bleomycin or in the kidney fibrosis induced by unilateral ureter obstruction, although they did contribute significantly to the liver fibrosis induced by CCl_4_ [35]. The significance of myofibroblasts in tissue fibrogenesis thus appears tissue-dependent [35]. It has been shown that fibrogenic cells in skeletal muscle are predominantly derived from resident PDGFRα^+^ mesenchymal progenitors [21, 36–39]. Myofibroblasts have been only identified in rare muscle diseases including nodular fasciitis and pseudomalignant myositis ossificans [40–42]. The significance of myofibroblasts in the muscle fibrosis associated with DMD is largely unknown [24]. Since targeting the α-SMA gene expression has been advocated for inhibiting the myofibroblast differentiation to treat tissue fibrosis [31], it is important to understand whether myofibroblast is a significant player in the skeletal muscle fibrosis associated with DMD, and whether targeting α-SMA can be useful in treating the muscular dystrophy. In the present study, we addressed the presence of myofibroblasts in dystrophic muscles of the *mdx*^*5cv*^ mice, and determined the correlation of α-SMA expression with the fibrogenic function of intramuscular fibrogenic cells.

## Materials and methods

### Animals

*Mdx*^*5cv*^ mice were derived from the Jackson laboratory. *ColI-GFP* transgenic mice with the green fluorescent protein (GFP) expression driven by collagen I (α1) gene promoter to label collagen I α1 producing cells with high sensitivity and specificity were kindly provided by Dr. David Brenner [43–45]. *Ccr2*^*-/-*^ mice were originally kindly provided by Dr. Israel Charo [46], which were backcrossed nine times with the C57BL/6J mice as previously described [47]. *Mdx*^*5cv*^ mice, instead of *mdx* mice, were used because the *mdx*^*5cv*^ mice are in the C57BL/6J background, same as the *Ccr2*^*-/-*^ mice and *ColI-GFP* mice, while the *mdx* mice are in a different background. The *mdx*^*5cv*^ mice display more severe functional deficits and similar muscle pathology as compared with the *mdx* mice [48]. *ColI-GFP/Mdx*^*5cv*^ and *ColI-GFP/Ccr2*^*-/-*^ mice were generated by crossbreeding the *ColI-GFP* mice with the *mdx*^*5cv*^ and *Ccr2*^*-/-*^ mice, respectively, for identifying and studying ColI (GFP)^+^ cells, which are mainly fibrogenic cells. All the study mice showed no gross abnormalities. Our study protocols were approved by the Institutional Animal Care and Use Committees at Icahn School of Medicine at Mount Sinai (New York, NY) and UT Southwestern Medical Center (Dallas, TX).

### Acute muscle injury

To induce acute skeletal muscle injury, 100 μl 1.2% barium chloride (BaCl_2_) was injected into the right quadriceps muscle of each wild-type (*WT*), *Ccr2*^*-/-*^, *ColI-GFP* or *ColI-GFP/Ccr2*^*-/-*^ mouse (age 10–14 weeks).

### Histopathological analysis

*WT*, *Ccr2*^*-/-*^, *ColI-GFP*, and *ColI-GFP/Ccr2*^*-/-*^ mice with acute muscle injury were sacrificed by CO2 asphyxiation and cervical dislocation at days 7 and 14 after BaCl_2_ injections. Male *mdx*^*5cv*^ and *ColI-GFP*/*Mdx*^*5cv*^ mice were sacrificed at 3 or 6 months. Quadriceps and diaphragm muscles were collected and fresh frozen in liquid nitrogen-cooled isopentane, sectioned at 8 μm, stained with hematoxylin and eosin, and viewed under a bright field microscope.

### Immunostaining

Immunostaining was performed using the protocols published by us [16, 49–51] and others [35]. Briefly, frozen muscle sections were fixed with 4% paraformaldehyde for 30 minutes, blocked in 5% serum for 2 hours, and incubated overnight with the primary collagen III antibody (Southern Biotech, Birmingham, AL, USA), GFP antibody (Invitrogen, Carlsbad, CA, USA), or Cy3-conjugated mouse anti-α-SMA antibody (Clone 1A4, Sigma-Aldrich, St. Louis, MO, USA) [35, 52]. For collagen III immunostaining, the sections were then washed with PBS and incubated with FITC-conjugated secondary antibody for 2 hours. After stained with DAPI, slides were mounted with mounting medium and antibody binding was visualized under a fluorescent microscope.

### Hydroxyproline assay

Muscles were snap-frozen and grounded in liquid nitrogen, followed by homogenization in distilled water. The muscle homogenates were then used for hydroxyproline assay using a kit purchased from Sigma-Aldrich (St. Louis, MO, USA) following the manufacturer's instruction.

### Single-cell suspension preparation and cell sorting

Muscle single-cell suspension was prepared by collagenase/dispase digestion [49, 50, 53]. Briefly, each muscle was minced in 2.5 ml of digestion solution containing 1U/ml of collagenase B and 1U/ml of dispase II (Roche Diagnostics, Indianapolis, IN, USA) in PBS and incubated at 37°C for 1 hour. The reaction was terminated by adding10 ml PBS with 10% FBS. The mixture was then filtered through a 70-μm cell strainer, and subjected to centrifugation twice at 250 g for 5 minutes. The pellets were combined, washed with PBS and centrifuged at 670 g for 10 minutes. The pellet was resuspended in 3 ml PBS, filtered through a 40-μm cell strainer, layered on equal volume of Lympholyte-M solution (Cedarlane, Burlington, NC, USA), and centrifuged at 2095 g for 45 minutes. Cells at the interface were collected, centrifuged at 670 g for 10 minutes, and resuspended in FACS staining buffer (PBS with 2% normal mouse serum (Invitrogen, Frederick, MA, USA) and 2% of BSA (Sigma, St. Louis, MO, USA)).

Cell sorting was performed by the Flow Cytometry Core of the Icahn School of Medicine at Mount Sinai and UT Southwestern Medical Center. Collagen I can be expressed by fibroblasts, fibro/adipogenic progenitor cells (FAP), satellite cells (CD45^-^/CD31^-^/Sca 1^-^/α7-integrin^+^), and fibrocytes (CD45^+^/Col1^+^) in fibrotic skeletal muscles [21, 39, 49, 54]. We used CD45^-^/CD31^-^/α7-integrin^-^/GFP (Col1)^+^ as a marker to identify and sort intramuscular fibrogenic cells. We used CD45^+^/F4/80^+^/Siglec F^-^ as a marker for intramuscular macrophages. We used the mice of the same genotype (*Ccr2*^*-/-*^ or *mdx*^*5cv*^) but without the GFP-Col1 transgene as negative controls to determine the cut-off for the GFP (Col1)^+^ cell population.

### Quantitative RT-PCR

Freshly sorted cells (1 million cells/sample) were lysed in the TRIzol reagent (Ambion, Grand Island, NY, USA). Total RNA was then purified and further cleaned up using the RNeasy Micro Kit (Qiagen, Hilden, Germany). Reverse transcription was performed using the SuperScriptTM II kit (Invitrogen, Frederick, MA, USA) following the manufacturer's instructions. The cDNA samples were then subjected to real-time PCR using the Sybr-green reagent and an Eppendorf Realpix4 cycler. The *gapdh* expression was used as an internal control. The reaction specificity was determined by product melting curves. The PCR products were verified by running 3% agarose gels. Data were analysed by ΔΔCt method and presented as Fold Changes. The following PCR primers were used: *col1α*, forward 5'-GCTCCTCTTAGGGGCCACT-3' and reverse 5'-CCACGTCTCACCATTGGGG-3'; *col3α*, forward 5'- AACCTGGTTTCTTCTCACCCTTC-3' and reverse 5'-CCACGTCTCACCATTGGGG-3'; *col6α*, forward 5'- CGCCCTTCCCACTGACAA-3' and reverse 5'- GCGTTCCCTTTAAGACAGTTGAG-3'; *fibronectin*, forward 5'- AAACTTGCATCTGGAGGCAAACCC-3' and reverse 5'- AGCTCTGATCAGCATGGACCACTT-3'; and α*-sma*, forward 5'- GACGTACAACTGGTATTGTG -3' and reverse 5'- TCAGGATCTTCATGAGGTAG -3'.

### Western blot

Whole cell extracts were prepared by lysing freshly sorted cells (0.3 million/sample) using RIPA buffer (Protein Simple, San Jose, CA, USA) supplemented with protease inhibitor cocktail (Thermal Scientific, Rockford, IL, USA). The extracts were subjected to Western blot analysis following the protocol previously published [16]. Antibodies with high sensitivity and specificity against β-actin and α-SMA were purchased from Cell Signalling Technology (Danvers, MA, USA).

### Statistical analyses

GraphPad (GraphPad software, Inc., La Jolla, CA, USA) was used for statistical analyses. All data were presented as mean ± SEM. Two-tailed Students *t* test was used when comparing two groups, and analysis of variance was performed with Bonferroni correction for multiple comparisons. A *p* value of <0.05 was considered statistically significant.

## Results

### α-SMA^+^/ColI (GFP)^+^ cells were not detected in the *ColI-GFP/mdx*^*5cv*^ diaphragm or quadriceps muscles by immunostaining

**Fig 1:**
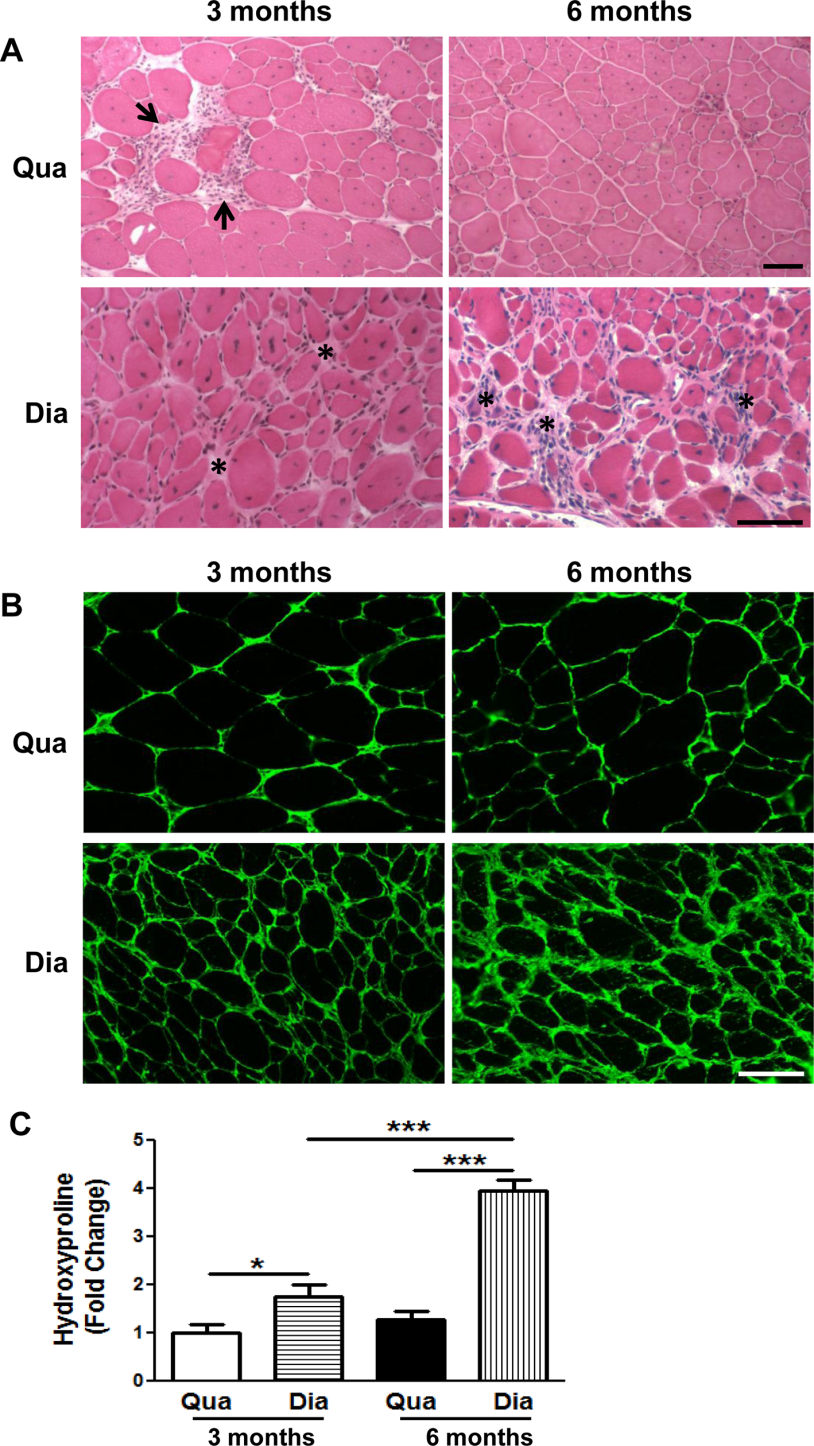
Fibrosis was more severe in diaphragm than in quadriceps muscles of *mdx*^*5cv*^ mice. (A) H&E staining of quadriceps (Qua) and diaphragm (Dia) muscles in the *mdx*^*5cv*^ mice at 3 months and 6 months of age (arrows: representative areas of myonecrosis and inflammation; asterisks: representative areas of endomysial fibrosis and inflammation). (B) Collagen III immunostaining of quadriceps (Qua) and diaphragm (Dia) muscles of *mdx*^*5cv*^ mice at 3 months and 6 months. (C) Hydroxyproline assay of quadriceps (Qua) and diaphragm (Dia) muscles from the *ColI-GFP/mdx*^*5cv*^ mice at 3 months and 6 months. Fold change refers to the comparison to the *mdx*^*5cv*^ quadriceps at 3 months. We used 5 mice/group/time point for tissue collections for HE staining, collagen III immunostaining, and protein preparation for hydroxyproline assay. *p<0.05, ***p<0.001. Bar = 50μm.

**Fig 2:**
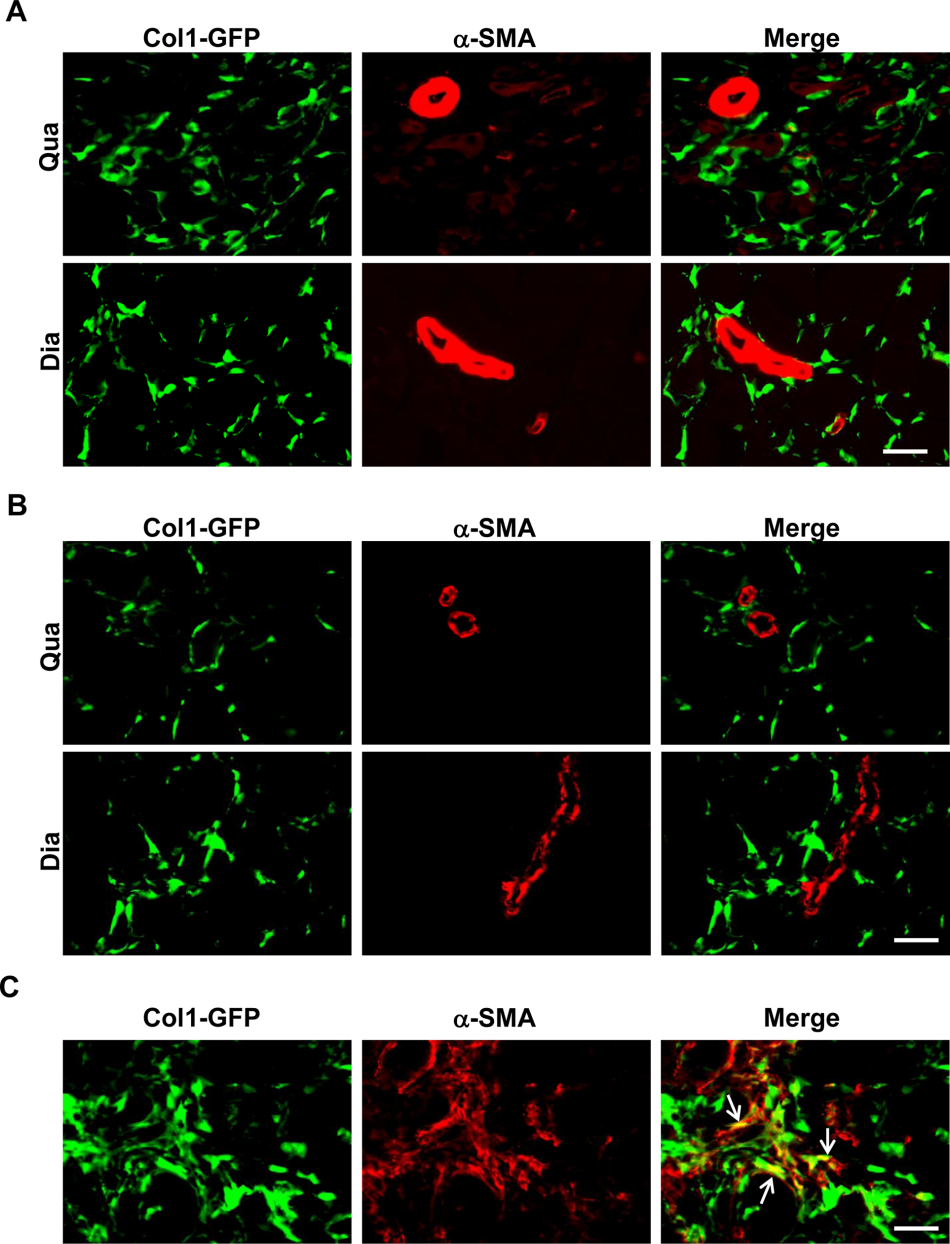
α-SMA^+^/ColI (GFP)^+^ cells were not detected in diaphragm or quadriceps muscles of *ColI-GFP/mdx*^*5cv*^ mice. (A) α-SMA/GFP (ColI) double immunostaining of quadriceps (Qua) and diaphragm (Dia) muscles in the *ColI-GFP/mdx*^*5cv*^ mice at 3 months. (B) α-SMA/GFP (ColI) double immunostaining of quadriceps (Qua) and diaphragm (Dia) muscles in the *ColI-GFP/mdx*^*5cv*^ mice at 6 months. (C) α-SMA/GFP (ColI) double immunostaining of fibrotic lungs from the *ColI-GFP* mice with the lung fibrosis induced by bleomycin (Lung). n = 5 mice/group. Bar = 50μm.

We first addressed whether myofibroblasts with co-expression of collagen I and α-SMA were detectable by GFP (ColI)/α-SMA double immunostaining in fibrotic muscles of the *ColI-GFP/mdx*^*5cv*^ mice. Endomysial fibrosis with mild thickening of endomysium was evident in both diaphragm and quadriceps muscles in the *mdx*^*5cv*^ mice at 3 months of age by H&E staining (Fig 1A) and collagen III immunostaining (Fig 1B). The H&E staining also showed multifocal and scattered myonecrosis and endomysial inflammation (Fig 1A). The fibrosis was more severe in the diaphragm than in the quadriceps as measured by hydroxyproline assay (Fig 1C). While the quadriceps fibrosis did not worsen at 6 months, the diaphragm fibrosis was progressive and became more severe at 6 months (Fig 1C). We performed GFP (ColI)/α-SMA double immunostaining in the diaphragm and quadriceps muscles of the *ColI-GFP/mdx*^*5cv*^ mice at 3 months (Fig 2A) and 6 months (Fig 2B). We used lung sections from the *ColI-GFP* mice with the lung fibrosis induced by bleomycin (kindly provided by Dr. Dean Sheppard) as a positive control (Fig 2C), because these sections were known to contain GFP (ColI)^+^/α-SMA^+^ myofibroblasts [35]. The double immunostaining showed many GFP (ColI)^+^ cells in the diaphragm and quadriceps muscles of the *ColI-GFP/mdx*^*5cv*^ mice at 3 months (Fig 2A) and 6 months (Fig 2B). It also showed high α-SMA staining signals in the smooth muscle cells lining intramuscular blood vessel walls (Fig 2A and 2B). While the GFP (ColI)^+^/α-SMA^+^ myofibroblasts were detected in the fibrotic lung (Fig 2C), they were not detected in the fibrotic diaphragm or quadriceps muscles (Fig 2A and 2B). Therefore, myofibroblasts with co-expression of collagen I and α-SMA are not detectable by immunostaining in the fibrotic muscles of the *mdx*^*5cv*^ mouse model of DMD.

### α-SMA^+^/ColI (GFP)^+^ cells were not detected in injured fibrotic muscles of *ColI-GFP/Ccr2*^*-/-*^ mice

**Fig 3:**
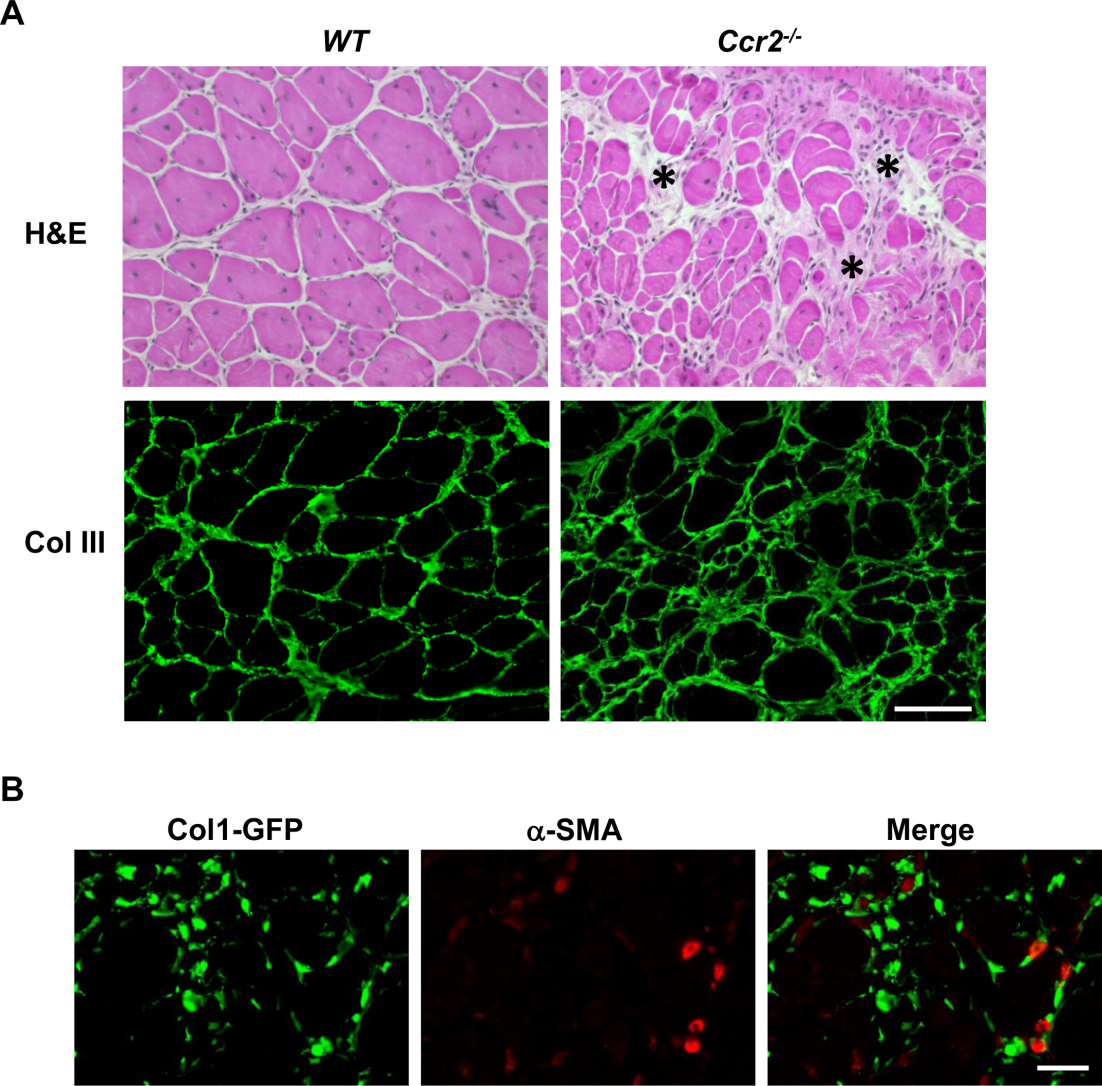
α-SMA^+^/ColI (GFP)^+^ cells were not detected in injured fibrotic muscles of *ColI-GFP/Ccr2*^*-/-*^ mice. (A) H&E staining and collagen III immunostaining of injured muscles at day 14 from the *WT* and *Ccr2*^*-/-*^ mice (asterisks: representative areas of endomysial fibrosis). (B) α-SMA/GFP (ColI) double immunostaining of injured muscles at day 14 from the *ColI-GFP/Ccr2*^*-/-*^ mice. n = 5 mice/group. Bar = 50μm.

We next addressed whether GFP (ColI)^+^/α-SMA^+^ myofibroblasts were detectable in fibrotic muscles with the fibrosis resulted from an etiology other than muscular dystrophy. To this end, we used acutely injured muscles induced by BaCl_2_ in the *ColI-GFP/Ccr2*^*-/-*^ mice. CC chemokine receptor type 2 (CCR2) mediates muscle recruitment of monocytes/macrophages (MOs/MPs), and the CCR2-mediated inflammation is essential to the acute skeletal muscle injury repair [24, 55–61]. Studies by our lab and others have shown that infiltrating MOs/MPs not only phagocytose damaged muscle fibers to allow tissue repair, but also produce myotrophic growth factors to promote muscle regeneration [57, 58]. Mice deficient in CCR2 display poor muscle regeneration with persistent muscle fibrosis following acute injury [56–59]. During normal acute skeletal muscle injury repair, there is transient muscle fibrosis with increased ECM protein deposition in endomysium, which provides a structural support for muscle repair. The transient fibrosis peaks at day 7, and then subsides [58]. While the endomysium of injured muscles at day 14 was minimally thickened in the wild-type (*WT*) mice, it was moderately thickened in the *Ccr2*^*-/-*^ mice as assessed by H&E staining and collagen III immunostaining (Fig 3A). The poor muscle regeneration in the *Ccr2*^*-/-*^ mice was evidenced by small regenerated fibers as compared with the WT controls (Fig 3A). We performed GFP (ColI)/α-SMA double immunostaining in injured muscles of the *ColI-GFP/Ccr2*^*-/-*^ mice at day 14. While the GFP (ColI)^+^ cells and α-SMA^+^ smooth muscle cells lining intramuscular blood vessel walls were readily detected, no GFP (ColI)^+^/α-SMA^+^ myofibroblasts were detectable (Fig 3B). Therefore, myofibroblasts are not detectable by immunostaining in fibrotic muscles with the muscle fibrosis caused by poor acute injury repair. Some α-SMA^+^ cells could be regenerating myofibers, as they could express α-SMA^+^ too [21].

### α-SMA gene and protein expression was detected by q-RT-PCR and Western blot in intramuscular ColI (GFP)^+^ fibrogenic cells of the *ColI-GFP/mdx*^*5cv*^ and *ColI-GFP/Ccr2*^*-/-*^ mice

**Fig 4:**
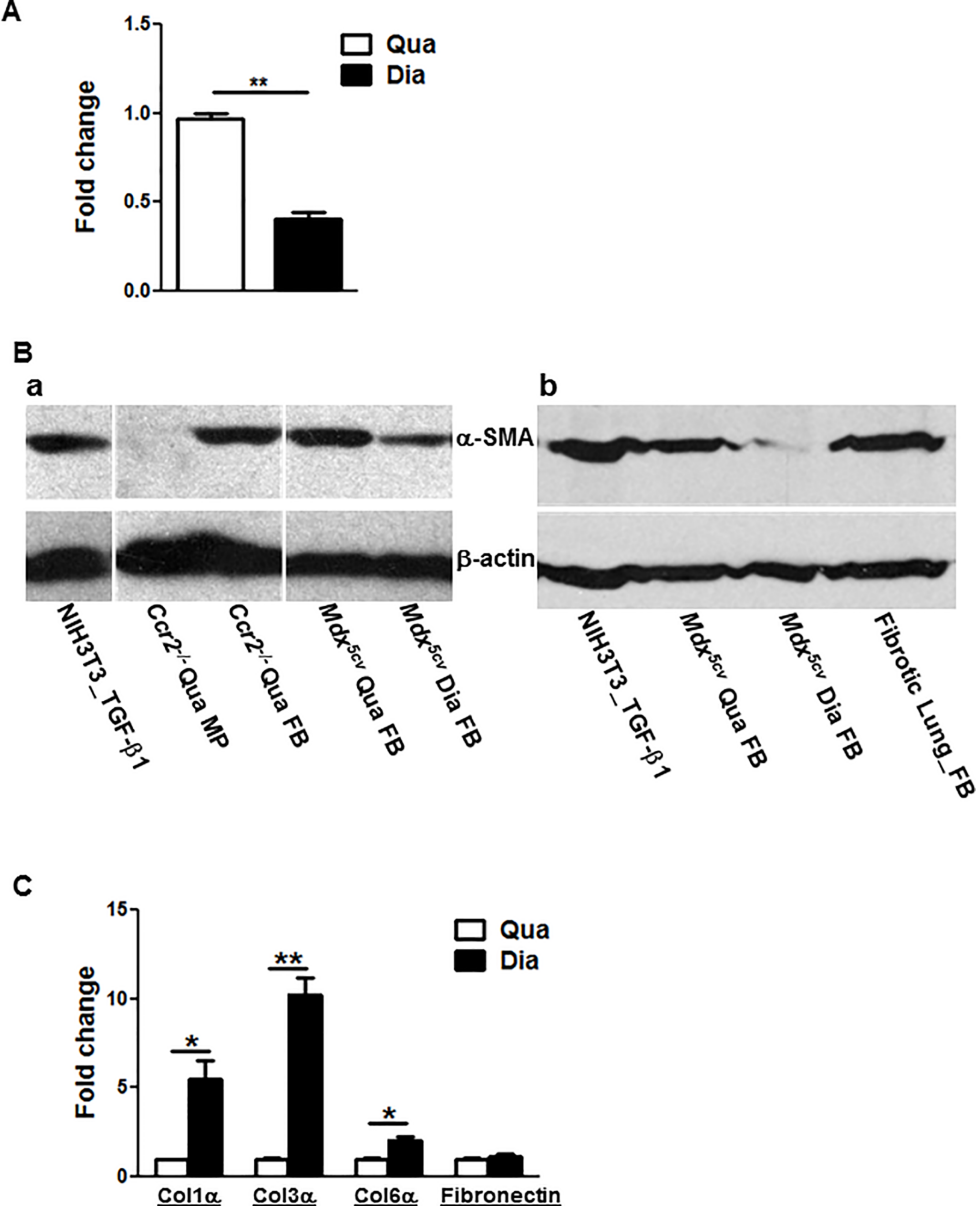
α-SMA gene and protein expression was detected in intramuscular CD45^-^/CD31^-^/α7-integrin^-^/GFP (Col1)^+^ fibrogenic cells. **The α-SMA expression level was low but the collagen expression level was high in the *ColI-GFP/mdx***^***5cv***^ **diaphragm fibrogenic cells as compared with the *ColI-GFP/mdx***^***5cv***^ **quadriceps fibrogenic cells. The α-SMA protein expression was low in the fibrogenic cells from *ColI-GFP/mdx***^***5cv***^ **diaphragm as compared with those from fibrotic lungs**. (A) qRT-PCR of α-SMA gene using fibrogenic cells freshly sorted from diaphragm (Dia) and quadriceps (Qua) muscles of the *ColI-GFP/mdx*^*5cv*^ mice at 3 months. (B) Western blot of α-SMA protein using fibrogenic cells (FB) freshly sorted from diaphragm (Dia) and quadriceps (Qua) muscles of the *ColI-GFP/mdx*^*5cv*^ (*mdx*^*5cv*^) mice at 3 months (a, b), from injured muscles at of the *ColI-GFP/Ccr2*^*-/-*^ mice (*Ccr2*^*-/-*^) day 7 post-injury (a), and from fibrotic lungs (Lung FB) with fibrosis induced by bleomycin in the *ColI-GFP* mice (b). NIH3T3 fibroblasts were used as a positive control, and intramuscular CD45^+^/F4/80^+^/Siglec F^-^ macrophages (MP) were used as a negative control. Data presented represents 3 independent experiments. (C) q-RT-PCR of ECM genes using fibrogenic cells freshly sorted from diaphragm (Dia) and quadriceps (Qua) muscles of the *ColI-GFP/mdx*^*5cv*^ mice at 3 months. Fold change refers to the comparison to the quadriceps fibrogenic cells of the *ColI-GFP/mdx*^*5cv*^ mice (A, C). Each experiment was independently performed twice. Each time, 10 mice were used for fibrogenic cell isolation and RNA or protein preparation. *p<0.05; **p<0.01.

To further address whether α-SMA was expressed by collagen-producing fibrogenic cells to represent myofibroblasts in fibrotic muscles, we performed qRT-PCR (Fig 4A) and Western blot (Fig 4B-a,b) using CD45^-^/CD31^-^/α7-integrin^-^/GFP (Col1)^+^ cells freshly sorted from the diaphragm and quadriceps muscles of the *ColI-GFP/mdx*^*5cv*^ mice at 3 months and from the acutely injured muscles of the *ColI-GFP/Ccr2*^*-/-*^ mice at day 7. We also used CD45^-^/CD31^-^/GFP (Col1)^+^ fibrogenic cells sorted from lungs with fibrosis induced by belomycin (Fig 4B-b). Both mRNA and protein expression of α-SMA was detected in the fibrogenic cells from these fibrotic muscles. The α-SMA expression level was lower in the *ColI-GFP/mdx*^*5cv*^ diaphragm fibrogenic cells than in the quadriceps fibrogenic cells (Fig 4A and 4B). This is in contrast to the more severe fibrosis seen in the diaphragm than in the quadriceps of the *ColI-GFP/mdx*^*5cv*^ mice (Fig 1). The level of α-SMA protein expression was low in the *ColI-GFP/mdx*^*5cv*^ diaphragm fibrogenic cells but was similar in the *ColI-GFP/mdx*^*5cv*^ quadriceps fibrogenic cells as compared to that in the fibrotic lung fibrogenic cells (Fig 4B-b).

### Collagen gene expression was higher in the diaphragm fibrogenic cells than in the quadriceps fibrogenic cells in the *ColI-GFP/mdx*^*5cv*^ mice

We next examined the fibrogenic function of the *ColI-GFP/mdx*^*5cv*^ diaphragm and quadriceps fibrogenic cells by their ECM gene expression. The mRNA expression of the collagen I, collagen III and collagen VI genes was higher in the diaphragm fibrogenic cells than in the quadriceps fibrogenic cells, and the mRNA expression of the fibronectin gene was similar (Fig 4C). Therefore, consistent with more severe fibrosis in the *ColI-GFP/mdx*^*5cv*^ diaphragm, the *ColI-GFP/mdx*^*5cv*^ diaphragm fibrogenic cells exert a higher fibrogenic function than the quadriceps fibrogenic cells despite a low level of α-SMA expression by the diaphragm fibrogenic cells. α-SMA is not a marker for the fibrogenic function of fibrogenic cells in dystrophic muscles.

## Discussion

Myofibroblasts are a subset of activated fibrogenic cells with enhanced expression of ECM proteins and neo-expression of α-SMA [26, 31]. They regulate connective tissue remodelling and play an important physiological role in wound repair. Myofibroblasts can also play a significant pathological role in tissue fibrosis by their enhanced ECM production and contractile force generation to contribute to the activation of integrin-bound latent TGF-β1 [32, 33]. There are no specific markers for myofibroblasts [24, 26, 31]. Myofibroblasts are conventionally identified by co-expression of collagen I and α-SMA [27–30], although α-SMA is also expressed by smooth muscle cells. α-SMA has been used not only as a differentiation marker but also as a functional marker for myofibroblasts [31–33]. While the origins, activation, differentiation, and effector functions of myofibroblasts have been characterized in fibrotic disease models of several tissues, including lung, liver, kidney, and skin [27, 30, 31], they have not been well-studied in the disease models of skeletal muscle fibrosis. By using the DMD mouse model, *ColI-GFP/mdx*^*5cv*^ mice, and the impaired acute skeletal muscle injury repair model, *ColI-GFP/Ccr2*^*-/-*^ mice, our present study shows that myofibroblasts with co-expression of collagen I and α-SMA are present in the fibrotic skeletal muscles, but their α-SMA expression is not detectable by immunostaining. The α-SMA expression level does not correlate positively with the fibrogenic function of intramuscular fibrogenic cells or the severity of muscle fibrosis. Therefore, α-SMA is not a functional marker of fibrogenic cells in skeletal muscle fibrosis associated with DMD.

Both α-SMA mRNA and protein are detectable by qRT-PCR and Western blot in collagen I-expressing cells purified from fibrotic muscles, which indicates that myofibroblasts are present in fibrotic muscles. However, the α-SMA^+^/ColI^+^ myofibroblasts are not detectable in fibrotic diaphragm or quadriceps in *ColI-GFP/mdx*^*5cv*^ mice by immunostaining despite the detection of many ColI^+^ cells and α-SMA^+^ smooth muscle cells lining intramuscular blood vessel walls. However, the α-SMA^+^/ColI^+^ myofibroblasts are detected in the lung sections with the fibrosis induced by bleomycin. Although the α-SMA protein expression level is low in the *ColI-GFP/mdx*^*5cv*^ diaphragm fibrogenic cells, it is similar in the *ColI-GFP/mdx*^*5cv*^ quadriceps fibrogenic cells when compared to the fibrogenic cells from the fibrotic lungs. The findings suggest that the α-SMA protein conformation in the myofibroblasts of the fibrotic muscles may be different from that in the myofibroblasts in the fibrotic lungs and in the smooth muscle cells in the fibrotic muscles. Both α-SMA protein expression level and protein conformation may contribute to the undetectable α-SMA by immunostaining in fibrotic muscles of the *ColI-GFP/mdx*^*5cv*^ mice. The α-SMA^+^/ColI^+^ cells are not detectable in the fibrotic muscles of the *ColI-GFP/Ccr2*^*-/-*^ mice either, suggesting that the lack of detectable myofibroblasts in fibrotic muscles by immunostaining is not specific to muscular dystrophy, as the muscle fibrosis in the *ColI-GFP/Ccr2*^*-/-*^ mice following acute injury is mainly caused by poor muscle regeneration [56, 58, 59]. It is unlikely that there is a technical issue with our immunostaining, because the α-SMA immunostaining signals are strong in smooth muscle cells lining intramuscular blood vessel walls, which serves as an internal positive control. The α-SMA immunostaining signals are also detected in some ColI^+^ cells in fibrotic lung sections, which serves as an external positive control. In addition, we used the same strong α-SMA antibody (clone 1A4 from Sigma) as used by other researchers, who detected α-SMA^+^/ColI^+^ myofibroblasts in fibrotic liver, lung, and kidney [35, 52].

Although α-SMA has been conventionally used as a differentiation and functional marker of tissue myofibroblasts, the importance of α-SMA and α-SMA-expressing myofibroblasts in tissue fibrogenesis varies among different tissues [35]. It has been reported that unlike the presence of many ColI^+^/α-SMA^+^ myofibroblasts in fibrotic liver with the fibrosis induced by CCl4, the α-SMA expression is detected by immunostaining only in a minority of collagen I-expressing cells in fibrotic lung and kidney with the fibrosis induced by bleomycin and unilateral ureter obstruction, respectively [35]. In the present study, we do not detect any cells with co-expression of collagen I and α-SMA in fibrotic muscles by immunostaining. This is consistent with the previous findings that myofibroblasts have been only identified in rare muscle diseases including nodular fasciitis and pseudomalignant myositis ossificans [40–42]. It has been shown that the α-SMA expression in myofibroblasts is not universally important in tissue fibrogenesis [35]. While fibroblasts with α-SMA expression did not contribute significantly to the lung fibrosis induced by bleomycin or the kidney fibrosis induced by unilateral ureter obstruction, they did contribute significantly to the liver fibrosis induced by CCl4, as the deletion of αv integrin in α-SMA-expressing cells protected against the experimental fibrosis in the liver but not in the lung or kidney [35]. Moreover, ColI^+^/α-SMA^+^ and ColI^+^/α-SMA^-^ fibroblasts from the fibrotic lung and kidney activated TGF-β equally [35]. These findings question the importance of α-SMA in the fibrogenic function of fibrogenic cells in lung and kidney, and strongly indicate that α-SMA is not a consistent functional marker of tissue fibrogenic cells [35]. The findings from our present study further support this notion. In contrast to the more severe fibrosis in the *ColI-GFP/mdx*^*5cv*^ diaphragm and the higher fibrogenic function of the *ColI-GFP/mdx*^*5cv*^ diaphragm fibrogenic cells, the α-SMA gene and protein expression levels are lower in the *ColI-GFP/mdx*^*5cv*^ diaphragm fibrogenic cells than in the *ColI-GFP/mdx*^*5cv*^ quadriceps fibrogenic cells. The findings demonstrate that α-SMA is not a functional marker of fibrogenic cells in skeletal muscle fibrosis associated with the muscular dystrophy.

In summary, myofibroblasts with co-expression of collagen I and α-SMA are present in fibrotic skeletal muscles, but they are not detectable by collagen I/α-SMA double immunostaining. α-SMA is not a marker of the fibrogenic function of intramuscular fibrogenic cells in the DMD mouse model. While targeting α-SMA gene expression has been advocated for inhibiting myofibroblast differentiation to reduce tissue fibrosis [31], this strategy may not be useful in treating skeletal muscle fibrosis associated with DMD.

## Supporting information

S1 Checklist Arrive Guideline Checklist. (PDF)

S1 Fig Fig 1C Hydroxyproline assay raw data. (XLSX)

S2 Fig Fig 4 qPCR raw data. (XLSX)

## Abbreviations

α-SMA: α-smooth muscle actin
CCR2: CC chemokine receptor type 2
ColI: collagen I
DGC: dystrophin-glycoprotein complex
DMD: Duchenne muscular dystrophy
ECM: extracellular matrix
MO: monocyte
MP: macrophage
TGF-β1: transforming growth factor-beta1
WT: wild-type
